# Comparison of metabolites in rumen fluid, urine, and feces of dairy cow from subacute ruminal acidosis model measured by proton nuclear magnetic resonance spectroscopy

**DOI:** 10.5713/ab.22.0124

**Published:** 2022-08-27

**Authors:** Hyun Sang Kim, Shin Ja Lee, Jun Sik Eom, Youyoung Choi, Seong Uk Jo, Jaemin Kim, Sang Suk Lee, Eun Tae Kim, Sung Sill Lee

**Affiliations:** 1 Institute of Agriculture and Life Science, Gyeongsang National University, Jinju 52828, Korea; 2 University-Centered Labs, Gyeongsang National University, Jinju 52828, Korea; 3 Division of Applied Life Science (BK21), Gyeongsang National University, Jinju 52828, Korea; 4 Ruminant Nutrition and Anaerobe Laboratory, College of Bio-industry Science, Sunchon National University, Suncheon 57922, Korea; 5 National Institute of Animal Science, Rural Development Administration, Cheonan 31000, Korea

**Keywords:** Feces, Fluid, Metabolite, Nuclear Magnetic Resonance (NMR), Ruminal Acidosis, Rumen, Urine

## Abstract

**Objective:**

In this study, metabolites that changed in the rumen fluid, urine and feces of dairy cows fed different feed ratios were investigated.

**Methods:**

Eight Holstein cows were used in this study. Rumen fluid, urine, and feces were collected from the normal concentrate diet (NCD) (Italian ryegrass 80%: concentrate 20% in the total feed) and high concentrate diet (HCD) groups (20%: 80%) of dairy cows. Metabolite analysis was performed using proton nuclear magnetic resonance (NMR) identification, and statistical analysis was performed using Chenomx NMR software 8.4 and Metaboanalyst 4.0.

**Results:**

The two groups of rumen fluid and urine samples were separated, and samples from the same group were aggregated together. On the other hand, the feces samples were not separated and showed similar tendencies between the two groups. In total, 160, 177, and 188 metabolites were identified in the rumen fluid, urine, and feces, respectively. The differential metabolites with low and high concentrations were 15 and 49, 14 and 16, and 2 and 2 in the rumen fluid, urine, and feces samples, in the NCD group.

**Conclusion:**

As HCD is related to rumen microbial changes, research on different metabolites such as glucuronate, acetylsalicylate, histidine, and O-Acetylcarnitine, which are related to bacterial degradation and metabolism, will need to be carried out in future studies along with microbial analysis. In urine, the identified metabolites, such as gallate, syringate, and vanillate can provide insight into microbial, metabolic, and feed parameters that cause changes depending on the feed rate. Additionally, it is thought that they can be used as potential biomarkers for further research on subacute ruminal acidosis.

## INTRODUCTION

The rumen environment is affected by many factors, such as diet type, feeding frequency, and the ratio of forage and concentrate [1]. In particular, the type of feed significantly affects rumen fermentation. The rumen microorganisms of ruminants decompose the feed and produce volatile fatty acid (VFA) as energy sources. In the forage fed, the buffering capacity of the diet is determined largely by the total chewing time, because the salivary buffer capacity of cows increases during chewing [2]. The effective fiber in forage is important in maintaining rumen function and stimulating the growth of fibrolytic bacteria, but the high content of indigestible fiber in corn stover has detrimental effects on nutrient utilization and digestibility, which depresses microbial protein synthesis and lowers nitrogen efficiency [3,4]. Compared to forage, concentrated feed consists of carbohydrates, such as starch, and can be rapidly decomposed in the rumen, resulting in a large amount of organic acid in the rumen, which can lead to acidosis.

The forage to concentrate ratio depends on the dietary ingredients and nutritional requirements of ruminant animals. Most previous studies have focused on the effects of different dietary forage to concentrate ratios on the bacterial community of Holstein heifers, energy utilization, growth rate, and feces microflora [5–7]. These processes are closely related to metabolic changes in the body. As cow health, growth, and metabolism are highly dependent on the production of metabolites in the rumen, a comprehensive analysis of the composition of rumen fluid can provide important insights into the role of rumen-diet interactions [8,9].

Previous studies of rumen on fed a high-concentrate diet have been observed in investigate feed efficiency, microbiota, and metabolites [10–12]. In a high concentrate diet (HCD), significantly increased the bacterial degradation product such as xanthine, uracil and amino acid such as leucine, glycine, and alanine in rumen metabolite. The relative abundance of cellulolytic bacteria and ciliates (*Succinimonas*, *Fibrobacter*, *Polyplastron*, and *Ostracodinium*) was linearly decreased according to increased concentrate diet level. Also, dairy cow on low pH in rumen had less backfat thickness and tended to have greater milk:feed ratio. Compared to the results of various studies on rumen, there are few studies on urine or feces that are relatively easy to sample in the field. Therefore, it is necessary to study of metabolite monitoring for change in various biofluids for dairy cows such as several previous studies [13,14].

We hypothesized that rumen acidosis caused by a HCD could be affect changes in urine and feces metabolite, and we thought that the difference could be used as research data for disease biomarkers. Therefore, the objective of this trial was to investigate the change of metabolites on the rumen fluid, urine, and feces of dairy cows brought about by different forage to concentrate ratios and exploration for potential biomarkers using proton nuclear magnetic resonance (NMR) analysis.

## MATERIALS AND METHODS

All experimental procedures including experimental animal maintenance and sample collection were conducted in accordance with guidelines from the committee of the National Institute of Animal Science, Rural Development Administration, Republic of Korea (Protocol Number: NIAS-2017-249). The study protocol was approved by the IACUC at National Institute of Animal Science.

### Animals and experimental diets

Eight Holstein cows (average body weight, 598±90 kg; parity, 2±1; 167±18 days in milk) from the National Institute of Animal Science were used in this study. For the trial, cows were classified according to feed ratio factors. The cows used in the experiment were divided into two groups: normal concentrate diet (NCD, n = 4) and HCD (n = 4). All cows were fed an NCD group diet (10 kg; Italian ryegrass 80%: concentrate 20%) and HCD group diet (14.2 kg; Italian ryegrass 20%: concentrate 80%) twice a day at 1000 h and 1600 h on a dry matter (DM) basis. The chemical composition of the feed is presented in Supplementary Table S1.

The contents of DM (method No. 934.01), crude protein (CP, method No. 976.05), calcium (method No. 927.02), and phosphorus (method No. 3964.06) in Italian ryegrass and concentrate were assayed as described by Association of Official Analytical Communities methods [15]. The contents of neutral detergent fiber and acid detergent fiber in Italian ryegrass and concentrate were assayed as described by Van Soest et al [16]. The rumen pH of HCD group and NCD group was monitored for 6 consecutive hours after feeding in the morning every day. The NCD group confirmed that the appropriate pH range was maintained and HCD group make sure the pH was below 5.5 for more than 3 h. The experiment lasted 14 days, with first 7 days as the diet adaptation period [17].

### Sample collection and preparation

Rumen fluid, urine, and feces samples were collected once for each animal, 4 h after the morning feeding on the last day of the experiment. Eight dairy cows were used for collection of rumen fluid through an oral stomach tube. The initially obtained rumen fluid (approximately 100 mL) was discarded to remove saliva and feed particle, and 200 mL of rumen fluid was collected in a conical tube. The pH of rumen fluid was analyzed immediately following collection using by pH meter (MP230, Mettler-Toledo, Columbus, OH, USA). Urine samples were collected after massage of the region underneath the vulva, in a 50 mL conical tube and immediately closed. Feces samples were collected directly through a clean anal area to the rectum of the animal in 50 mL conical tube. The collected samples were stored at −80°C until analysis.

The rumen fluid sample was thawed at 4°C and centrifuged at 12,902×g for 10 min to collect 300 μL of the supernatant. The reference material 2,2,3,3-d(4)-3-(Trimethylsilyl)propionic acid sodium salt (TSP) was dissolved in deuterium oxide (D_2_O) to make 300 μL of 0.4 mM and added with the prepared rumen fluid sample to make the 0.2 mM TSP concentration in the total mixed solution. The preparation of the rumen fluid sample was described elsewhere [14].

Urine samples added to 0.2 M sodium phosphate buffer (pH 7.0) were centrifuged at 14,000×g at 4°C for 10 min to collect 400 μL of supernatant. The supernatant was added to 230 μL of buffer and the pH was adjusted to 7.0±0.1. The 540 μL mixture was added to 5 mM TSP (60 μL) in D_2_O and the 0.5 mM TSP concentration of the 600 μL total solution. The preparation of urine samples was described elsewhere [14].

The feces sample thawed at 4°C was centrifuged at 14,000×g for 20 min to collect of the supernatant. The supernatant was recentrifuged at 12,902×g for 10 min and 400 μL sample was added to 140 μL sodium phosphate buffer (pH 7.0). The reference material TSP was dissolved in D_2_O to make 60 μL of 5 mM and added with the prepared fecal sample to make the 0.5 mM TSP concentration in the total mixed solution. The preparation of the fecal sample was described elsewhere [14].

### Proton NMR analyses

The proton NMR spectra were acquired using AVANCE III HD Bruker spectrometer (Bruker BioSpin AG, Fallanden, Switzerland). To acquire spectra of the rumen, urine, and feces samples, the Bruker standard nuclear overhauser enhancement spectroscopy (NOESY) pre-saturation pulse sequence was used with the following parameters: temperature = 25°C, repetition number = 128, acquisition time = 2.0 s. The FID was acquired with a spectral width of 20 ppm and collected to 64k data points.

### The NMR data processing and statistical analyses

Metabolite qualitative and quantification were carried out by importing the analyzed spectral data into the Chenomx NMR suite 8.4 software (ChenomxInc, Edmonton, Canada). The spectra were manually baseline and phase using Chenomx processor 8.4 software. The spectral width was 10 ppm and was referenced to the TSP signal at 0 ppm. The metabolite databases used for classification were the bovine metabolome database (www.bovinedb.ca), livestock metabolome database (www.lmdb.ca), and the human metabolome database (www.hmdb.ca).

All metabolite data analyses were performed using by Metaboanalyst 4.0 (www.metaboanalyst.ca) [18]. The resulting data were normalization selected methods follow as raw wise normalization: normalization to constant sum; data transformation: Log10 normalization; data scaling: pareto scaling.

The between-group differential metabolites were analyzed using student’s t-test (p<0.05). The multivariate data analysis was performed principal component analysis (PCA), partial least squares-discriminant analysis (PLS-DA), orthogonal partial least squares-discriminant analysis (OPLS-DA) and variable importance in projection (VIP) was created based on PLS-DA results.

## RESULTS

### Metabolite profiles of rumen fluid, urine and feces

**Figure 1:**
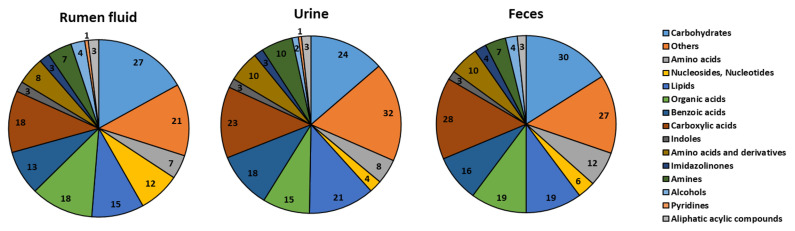
The classification of metabolite on rumen fluid, urine, and feces to the detectable metabolome. Identified metabolites are categorized according to chemical class (bovine metabolome database and livestock metabolome database) and the number of metabolites detected by proton nuclear magnetic resonance.

The classification of metabolites is shown in Figure 1. Original concentration of rumen fluid, urine, and feces are provided as supplementary information (Supplementary Tables S2–4). One hundred sixty metabolites were identified in the rumen fluid (four alcohols, three aliphatic acyclic compounds, seven amines, 15 amino acids, 13 benzoic acids, 27 carbohydrates, 18 carboxylic acids, three imidazolinones, three indoles, 15 lipids, 12 nucleosides and nucleotides, 18 organic acids, 21 others, and one pyridine).

In the urine samples, 177 metabolites (two alcohols, three aliphatic acyclic compounds, 10 amines, 18 amino acids, 18 benzoic acids, 24 carbohydrates, 23 carboxylic acids, three imidazolinones, three indoles, 21 lipids, four nucleosides and nucleotides, 15 organic acids, 32 others, and one pyridine) were identified.

In the feces samples, 188 metabolites (four alcohols, three aliphatic acylic compounds, seven amines, 22 amino acids, 16 benzoic acids, 30 carbohydrates, 28 carboxylic acids, four imidazolinones, three indoles, 19 lipids, six nucleosides and nucleotides, 19 organic acids, and 27 others) were identified.

### Multivariate data analysis

**Figure 2:**
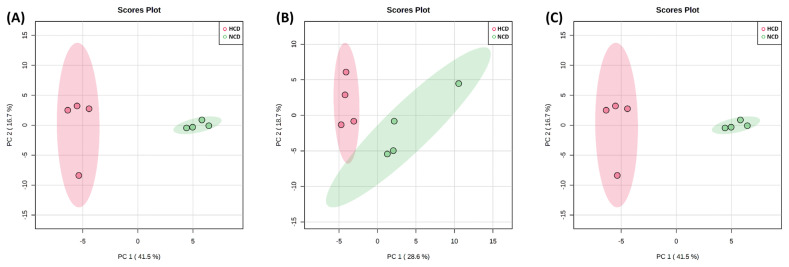
Principal component analysis (PCA) of the (A) rumen fluid, (B) urine, and (C) feces metabolite. The PCA plot distinguishes the metabolic profiles in cows fed diets based on high concentrate diet (HCD, red dots) vs normal concentrate diet (NCD, green dots). Ellipse represents 95% confidence interval.

The PCA score plot shows that the model interpretation rates for the HCD group and NCD group (Figure 2). The PCA revealed that PC 1 and 2 accounted for 41.5% and 16.7%, 28.6% and 18.7%, and 29.3% and 21.1% of the total variation in the rumen fluid, urine, and feces, respectively. The two groups of rumen fluid and urine samples were well separated, and samples from the same group were well aggregated together. On the other hand, the feces samples were not separated and showed similar tendencies between the two groups.

**Figure 3:**
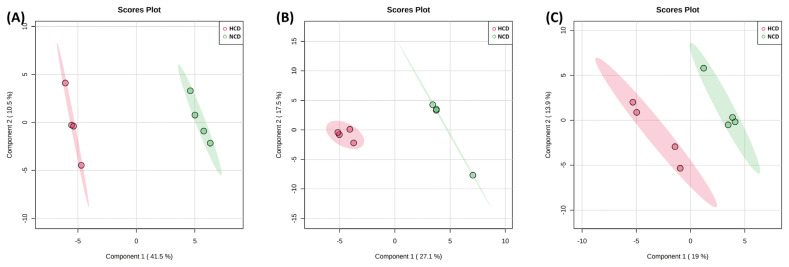
Partial least squares-discriminant analysis (PLS-DA) of the (A) rumen fluid, (B) urine, and (C) feces metabolite. The PLS-DA plot distinguishes the metabolic profiles in cows fed diets based on high concentrate diet (HCD, red dots) vs normal concentrate diet (NCD, green dots). Ellipse represents 95% confidence interval.

The PLS-DA is a versatile algorithm that can be used for predictive and descriptive modeling as well as for discriminative variable selection (Figure 3). In the score plot of PLS-DA, the NCD and HCD groups were discriminated with an R^2^ of 0.988, a Q^2^ of 0.870 in rumen fluid. In urine and feces was 0.940 and 0.551 and 0.791 and −0.6372, respectively. The R^2^ (coefficient of determination) explains how well the model fits the data, and R^2^ close to one is one of the necessary conditions for the model to be robust. The value of Q^2^ (cumulative prediction ability) indicates the ability of the model to predict new data, and a larger Q^2^ indicates that the model has good predictive performance.

**Figure 4:**
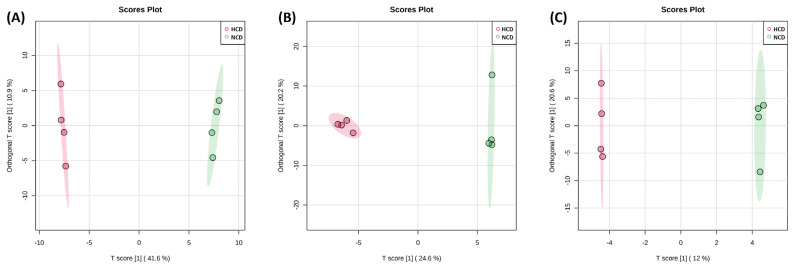
Orthogonal partial least squares-discriminant analysis (OPLS-DA) of the (A) rumen fluid, (B) urine, and (C) feces metabolite. The OPLS-DA plot distinguishes the metabolic profiles in cows fed diets based on high concentrate diet (HCD, red dots) vs normal concentrate diet (NCD, green dots). Ellipse represents 95% confidence interval.

**Figure 5:**
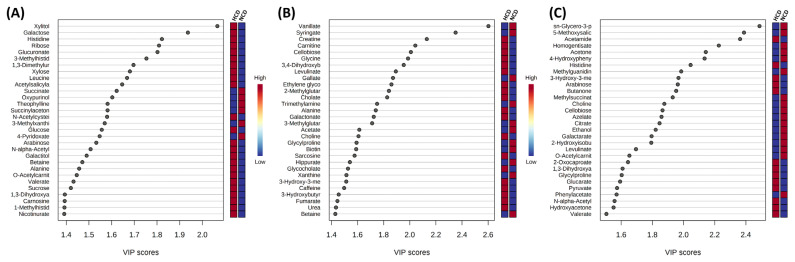
The top 30 metabolites of variable importance in projection (VIP) scores of metabolite in (A) rumen fluid, (B) urine, and (C) feces in cows fed diets based on high concentrate diet (HCD) vs normal concentrate diet (NCD). The selected metabolite were those with VIP>1.0 based on the parial least squares-discriminant analysis model. The red and blue colors of the heat map on the right indicate the abundance of each metabolite from high to low.

An OPLS-DA supervised model was used to assess intergroup differences and permutation test (set permutation number 1,000) for statistical testing under the null hypothesis. In the rumen fluid and urine, R^2^Y, and Q^2^ were 0.999 and 0.947 and 0.999 and 0.792, respectively, whereas in the feces, R^2^Y = 0.489 and Q^2^ = 0.139 (Figure 4). Both the R^2^Y and Q^2^ values were greater than 0.4, indicating that the model was stable and reliable, except for the feces samples. A Q^2^ value of approximately 1 indicated that the OPLS-DA model had good predictability. The p-values of permutation for rumen fluid were 0.029 (R^2^Y) and 0.029 (Q^2^). In the p-value of urine and feces, R^2^Y and Q^2^ were 0.061 and 0.031 and 0.501 and 1. The VIP score >1.0 metabolite, and heat map of VIP scores are shown in Figure 5.

### Differential metabolite analysis

A univariate analysis using a t-test was conducted for rumen, urine, and feces metabolites for the NCD group and HCD group. The results of significantly (p<0.05) lower and higher metabolites between the two groups are shown in Tables 1 and 2, respectively. We identified a total of 64 differential metabolites in the rumen fluid, of which 15 had low concentrations and 49 had high concentrations in the HCD group compared to the NCD group. In the urine and feces samples, the metabolites with low and high concentrations were 14 and 16, and 2 and 2, respectively.

## DISCUSSION

The rumen plays a central role in the efficiency of digestion in ruminants. The pattern of rumen metabolites shows the degradation products of rumen microorganisms, depending on the feed ratio. Non-fiber carbohydrates (NFCs), including starch, monosaccharides, oligosaccharides, and pectin, are rapidly decomposed by rumen microorganisms to produce VFAs [19]. A higher NFC ratio in feed increases the production of VFAs, resulting in a decrease in rumen pH [20]. Subacute rumen acidosis occurs when the rumen pH remains low between 5.8 and 5.5 at least 3 h/d [17]. Subacute ruminal acidosis leads to the destruction of bacteria composition balance due to the decrease in ruminal pH sensitive cellulolytic bacteria and the dominance of acid-resistant bacteria. The risk of ruminal acidosis causes various effects of economic loss such as low milk production and laminitis to endotoxin released from *Streptococcus bovis* [21].

In this study, the NFC content of NCD and HCD groups was 28.0% and 45.8% (DM %), respectively. According to a study by AlZahal et al [22], if the NFC content of the feed exceeds 40% of the DM standard, it is possible to reduce the rumen pH to less than 5.6 for 5.0 h/d.

The PCA and PLS-DA analyses showed a clear separation between rumen fluid and urine metabolites, except for feces metabolites, owing to the different diets offered in the present study, thereby representing a significant difference in metabolic changes.

In particular, metabolites related to carbohydrates and amino acids were significantly altered. We know that the feed ratio of the HCD group contained a lot of starch and polysaccharides were rapidly decomposed into glucose in the rumen. Among the carbohydrate metabolites, those that were significantly higher in the HCD group were glucose, arabinose, ribose, glucoronate, and xylose. In particular, xylose and arabinose account for up to 80% of the carbohydrates that comprise the hemicellulose of forage and cereals [23]. This is attributed to the fact that there are more soluble components in the concentrated feed. Glucuronate is a sugar acid derived from glucose, and its sixth carbon atom is oxidized to a carboxylic acid [24]. Glucuronate is a xylan degradation product and a source of pyruvate necessary for metabolic processes. In particular, glucuronate is an important growth substrate for b316, which plays a key role in the degradation of plant polysaccharides, producing butyrate as an end-product [25]. Leucine is an essential amino acid for mammals as a substrate for protein synthesis and is considered an efficient nutrient signal that regulates protein synthesis [26]. Histidine is an essential amino acid that cannot be metabolically synthesized and must therefore be absorbed from the diet. Histidine is used through bacterial metabolism to degrade glutamate, ammonia, and single carbon compounds [27]. 3-Methylhistidine is a metabolite of protein and energy metabolism, increased levels of which are observed in the rumen when the animal is fed a diet high in rumen-degradable protein [28]. In blood and urine, 3-Methylhistidine levels are used as biomarkers of skeletal muscle degradation and muscle protein turnover in animals [29]. Succinate was significantly higher in the NCD group, which had a high rate of forage. Many pure cultures of rumen bacteria form succinate as a final fermentation product in the rumen; however, this does not accumulate in the rumen [30]. Added succinate is rapidly converted to propionate in the rumen by washed rumen bacteria [31].

Urine is a liquid that excretes water-soluble by-products produced through metabolic pathways in animals. It has much to do with nitrogen circulation in the body. Urea is produced in the liver by the ureagenesis of ammonia, which is absorbed through the rumen wall. Colmenero et al [32] reported that the urinary level of urea is affected by the CP content of the feed. The increase in urea in the HCD group was similar to that reported in previous studies wherein the amount of urea-N excreted increased with the increase in the CP intake, and the correlation between the two was confirmed [33]. Amino acids, including alanine and glycine are produced by transamination in muscle and other tissues, and transport nitrogen for urea production through amino acid catabolism in the liver. The amino acid net flux of the liver represents the sum of unidirectional removal and release, and in the case of amino acids that can be released from the liver, the indication of unidirectional removal may be greater than the measured value of the net flux [34]. The relationship between the alanine and the high-concentrate diet groups observed in this study can be explained. Trimethylamine-N-oxide is synthesized from the oxidation of trimethylamine and is correlated with dietary choline. Soy, whole grains, and vegetables are sources of choline. Interestingly, our results showed that the concentration of forage-derived phenolic compounds (such as vanillate, syringate, gallate, and hippurate) was high in the NCD group. However, there was no significant difference between the groups. The anaerobic degradation of lignin-derived aromatic metabolites has been studied in various research fields [35]. Consequently, we confirmed the existence of gallate, vanillate, and syringate as degradation intermediates and lignin-derived methoxylated monoaromatics. The observed high values in three metabolites (vanillate, syringate, and gallate) could be interpreted as being a result of feeding a high forage ratio. Hippurate is a metabolite commonly identified in the urine of mammals and is related to the intake of phenolic compounds. Phenylalanine, chlorogenic acid, and catechin are metabolized to produce benzoic acid, which is then transferred to the mitochondria to produce hippurate [36]. Creatine plays an important role in the energy response through its conversion creatine phosphate via phosphorylation related to energy transfer, generating new ATP [35]. Creatine can accumulate in the body of animals through de novo synthesis and diet [37], and is mainly synthesized in the liver using glycine, arginine, and methionine, and transferred to tissue through the blood. Residual creatine is excreted through urine. It is thought to be significantly higher in HCD group than in NCD group due to the excretion of creatine, which is not used as an energy source in the body due to the high feeding of concentrated feed. Carnitine is required for the transport of long-chain fatty acids from the cytoplasm to the matrix compartment of the mitochondria for energy production, and is also involved in a variety of important physiological processes, including ketogenesis, thermogenesis, lipolysis, and potential nitrogen metabolism [38]. The urinary carnitine concentration was eight times higher in the HCD group than in the NCD group. Urinary carnitine is derived from the blood and can be used as a predictor of a high concentration of fatty acids that must be used in the body. The choline in feed is degraded by rumen microorganisms by approximately 80%, and metabolic choline is supplied by endogenous synthesis via the phosphatidylethanolamine N-methyltransferase pathway [39].

In this study, metabolites in the rumen fluid, urine, and feces of dairy cows with different feed ratios were screened as non-targets using proton NMR. The metabolites in the rumen fluid changed according to the feed rate. These included glucose, xylose, and maltose related to carbohydrate metabolism, and leucine, alanine, and isoleucine related to protein metabolism, which was found to be highly concentration in the HCD group. As HCD group is related to rumen microbial changes, research on different metabolites such as glucuronate, acetylsalicylate, histidine, and O-Acetylcarnitine, which are related to bacterial degradation and metabolism, will need to be carried out in future studies along with microbial analysis. In urine, muscle metabolism and microbial-derived metabolites such as creatine, alanine, and xanthine were identified. In addition, the identified metabolites, such as gallate, syringate, and vanillate originated from the NCD group. These metabolites can provide insight into microbial, metabolic, and feed parameters that cause changes depending on the feed rate; additionally, it is thought that they can be used as potential biomarkers for further research.

## Figures and Tables

**Table 1:** The statistically low metabolites in high concentrate diet (HCD) compared to normal concentrate diet (NCD)

Metabolite^1)^	FC^2)^	p-value	VIP^3)^	Metabolite	FC	p-value	VIP
Rumen fluid							
Methylamine	0.34	0.047	1.29	Oxypurinol	0.08	<0.001	1.60
Succinate	0.08	<0.001	1.62	4-Pyridoxate	0.11	<0.001	1.54
UDP-N-Acetylglucosamine	0.23	0.030	1.00	1,7-Dimethylxanthine	0.14	0.005	1.29
Succinylacetone	0.08	<0.001	1.58	Ibuprofen	0.22	0.042	0.98
Ferulate	0.14	0.014	1.23	3-Phenylpropionate	0.38	0.003	0.98
Gluconate	0.18	0.050	1.01	Acetate	0.70	<0.001	0.61
Urine							
Methanol	0.31	0.039	1.42	3-Methylglutarate	0.19	0.014	1.71
Trimethylamine N-oxide	0.17	<0.001	1.74	Thymol	0.34	0.019	1.27
Hippurate	0.25	<0.001	1.54	Xanthine	0.25	<0.001	1.51
Vanillate	0.02	<0.001	2.60	Succinylacetone	0.33	0.034	1.16
Gallate	0.12	<0.001	1.87	Betaine	0.13	0.049	1.42
Syringate	0.16	0.021	2.35	N-Methylhydantoin	0.42	0.008	1.15
Glycylproline	0.21	0.001	1.59	trans-Aconitate	0.40	0.006	1.15
Feces							
5-Methoxysalicylate	0.19	0.045	2.38	4-Hydroxyphenyllactate	0.20	0.016	2.13

1) The metabolite was significant at p-value<0.05 by the t-test

2) FC, fold change was calculated by dividing the value of metabolites in HCD group by partial NCD group.

3) VIP, variable importance in projection were obtainded from partial least squares-discriminant analysis model.

**Table 2:** The statistically high metabolites in high concentrate diet (HCD) compared to normal concentrate diet (NCD)

Metabolite^1)^	FC^2)^	p-value	VIP^3)^	Metabolite	FC	p-value	VIP
Rumen fluid							
Isopropanol	3.87	0.024	1.20	Glycylproline	3.31	0.042	1.01
Kynurenine	2.57	0.013	0.89	3-Hydroxyphenylacetate	3.53	0.011	1.10
Carnosine	5.77	0.007	1.39	3-Hydroxyisovalerate	3.73	0.009	1.15
Anserine	4.59	0.002	1.27	N-Carbamoylaspartate	4.00	0.007	1.23
1-Methylhistidine	7.32	0.003	1.39	cis-Aconitate	5.22	0.004	1.35
3-Methylhistidine	15.94	0.001	1.75	Homovanillate	6.08	0.007	1.39
Histidine	18.85	<0.001	1.82	N-Acetylcysteine	8.51	0.004	1.58
Isoleucine	5.05	0.001	1.26	N-alpha-Acetyllysine	8.57	0.004	1.50
Alanine	6.42	0.004	1.45	5-Hydroxyindole-3-acetate	3.27	0.003	1.11
Leucine	11.63	0.001	1.66	3-Hydroxy-3-methylglutarate	3.12	0.020	1.03
4-Hydroxyphenylacetate	2.84	0.001	1.04	O-Acetylcarnitine	9.96	0.033	1.44
3,4-Dihydroxybenzeneacetate	3.91	<0.001	1.21	NAD	4.71	<0.001	1.25
o-Cresol	4.74	0.023	1.32	Butyrate	1.57	<0.001	0.69
3-Hydroxymandelate	5.01	<0.001	1.32	N-Nitrosodimethylamine	2.93	0.015	1.03
Acetylsalicylate	10.72	0.002	1.64	Salicylate	3.83	<0.001	1.20
Maltose	5.64	0.002	1.30	Nicotinate	4.63	<0.001	1.29
1,3-Dihydroxyacetone	5.93	<0.001	1.39	Phenylacetate	5.17	0.003	1.24
Glucose	8.93	<0.001	1.55	Nicotinurate	6.19	<0.001	1.39
Galactitol	9.71	0.001	1.48	Valerate	6.63	0.004	1.43
Arabinose	9.84	0.003	1.53	2-Hydroxyphenylacetate	3.87	0.029	0.98
Ribose	17.27	0.001	1.80	Desaminotyrosine	4.16	<0.001	1.24
Glucuronate	18.33	<0.001	1.80	Caffeine	4.18	0.018	1.21
Xylose	22.69	0.006	1.68	Betaine	5.96	0.013	1.47
Galactose	34.71	<0.001	1.93	1,3-Dimethylurate	17.57	0.003	1.69
Xylitol	46.96	<0.001	2.06				
Urine							
Urea	3.24	<0.001	1.43	Hydroxyacetone	2.52	0.047	1.35
Sarcosine	4.19	0.002	1.57	Choline	4.88	0.027	1.60
N-Phenylacetylglycine	3.14	0.037	1.39	Cholate	5.30	0.030	1.82
Creatine	48.02	0.029	2.13	2-Methylglutarate	6.00	0.014	1.84
Alanine	5.88	<0.001	1.73	Carnitine	8.58	0.006	2.04
Glycine	27.40	0.023	1.98	Ethylene glycol	9.56	0.006	1.85
3,4-Dihydroxybenzeneacetate	9.79	0.002	1.95	Levulinate	10.42	0.003	1.89
Galactonate	6.70	0.025	1.72	Cellobiose	7.49	0.016	2.00
Feces							
Histidine	4.51	0.020	2.04	2-Oxocaproate	2.82	0.050	1.64

1) The metabolite was significant at p-value<0.05 by t-test.

2) FC, fold change was calculated by dividing the value of metabolites in HCD group by partial NCD group.

3) VIP, variable importance in projection were obtainded from partial least squares-discriminant analysis model.

## SUPPLEMENTARY MATERIAL

**Supplementary Table S1**. The formulation and chemical composition of experiment diet

**Supplementary Table S2**. Concentrations of the rumen fluid metabolite by ^1^H-NMR analysis (μM, Median±interquartile range, n = 4)

**Supplementary Table S3**. Concentrations of the urine metabolite by ^1^H-NMR analysis (μM, Median±interquartile range, n = 4)

**Supplementary Table S4**. Concentrations of the feces metabolite by 1H-NMR analysis (μM, Median±interquartile range, n = 4)
