# Supplementary material for: Comparison of metabolites in rumen fluid, urine, and feces of dairy cow from subacute ruminal acidosis model measured by proton nuclear magnetic resonance spectroscopy
Source: Anim Biosci. 2022 Aug 27;36(1):53–62. doi: 10.5713/ab.22.0124 (PMC9834661; doi:10.5713/ab.22.0124)
Supplement: Supplementary file 1 [file ab-22-0124-suppl1.pdf]

**Supplementary table 1.** The formulation and chemical composition of experiment diet

| Content | *% of DM* | Content | Concentrate | Italian ryegrass |
|---|---|---|---|---|
| *Ingredients* | | *Chemical composition (% of DM)* | | |
| Ground corn | 3.06 | Moisture | 11.20 | 7.40 |
| Ground wheat | 17.44 | Crude protein | 20.01 | 5.60 |
| Salt | 0.70 | Ether extract | 2.00 | 0.84 |
| Molasses | 3.50 | Crude ash | 6.43 | 4.84 |
| Soy hull | 5.36 | Crude fiber | 20.00 | 34.84 |
| Wheat flour | 20.10 | Ca | 0.80 | 0.21 |
| Rice bran | 0.84 | P | 0.51 | 0.13 |
| Corn DDG | 5.00 | Acid detergent fiber | 6.86 | 38.95 |
| DDGS | 15.00 | Neutral detergent fiber | 19.81 | 66.54 |
| Soybean meal | 6.12 | | | |
| Urea | 0.51 | | | |
| Sodium bicarbonate | 0.84 | | | |
| Condensed molasses solubles | 1.50 | | | |
| Corn gluten feed | 18.00 | | | |
| Limestone | 1.27 | | | |
| Palm oil | 0.39 | | | |
| Flavor | 0.02 | | | |
| Mineral/Vitamin premix[1] | 0.35 | | | |
| Total | 100.00 | | | |

[1]Mineral & vitamin premix contained vit. A 2,650,000 IU, vit. D3 530,000 IU, vit. E 1,050 IU, niacin 10,000 mg, Mn 4,400 mg, Zn 4,400 mg, Fe 13,200 mg, Cu 2,200 mg, iodine 440 mg, and Co, 440 mg/kg of Grobic-DC provided from Bayer Health Care (Leverkusen, Germany).

DDG: Distillers dried grains, DDGS: Distillers dried grains with solubles.
