# Supplementary material for: Comparison of metabolites in rumen fluid, urine, and feces of dairy cow from subacute ruminal acidosis model measured by proton nuclear magnetic resonance spectroscopy
Source: Anim Biosci. 2022 Aug 27;36(1):53–62. doi: 10.5713/ab.22.0124 (PMC9834661; doi:10.5713/ab.22.0124)
Supplement: Supplementary file 2 [file ab-22-0124-suppl2.pdf]

Supplementary table 2. Concentrations of the rumen fluid metabolite by ^{1}H-NMR analysis (μM, Median ± interquartile range, n = 4)

No.	Metabolite	NCD[1)]	HCD[2)]
1	1,3-Dihydroxyacetone	6.15 ± 2.65	53.95 ± 11.48
2	1,3-Dimethylurate	0.48 ± 0.48	11.9 ± 6.51
3	1,7-Dimethylxanthine	2.42 ± 1.41	0.47 ± 0.06
4	2'-Deoxyadenosine	0.79 ± 0.03	4.25 ± 2.86
5	2'-Deoxyuridine	3.26 ± 3.43	3.51 ± 3.57
6	2-Furoylglycine	0.43 ± 0.06	3.01 ± 2.33
7	2-Hydroxy-3-methylvalerate	13.39 ± 13.69	45.19 ± 48.8
8	2-Hydroxyisocaproate	15.76 ± 15.76	60.71 ± 105.66
9	2-Hydroxyphenylacetate	3.55 ± 0.41	19.65 ± 12.29
10	2-Oxoisocaproate	0.56 ± 0.56	2.01 ± 3.46
11	3,4-Dihydroxybenzeneacetate	7.55 ± 0.25	44.5 ± 8.29
12	3,5-Dibromotyrosine	5.77 ± 3.46	1.76 ± 1.76
13	3-Hydroxy-3-methylglutarate	21.8 ± 11.93	96.15 ± 30.72
14	3-Hydroxybutyrate	18.93 ± 20.02	7.34 ± 11.24
15	3-Hydroxyisovalerate	9.2 ± 5.25	47.9 ± 9.35
16	3-Hydroxykynurenine	5.87 ± 6.39	2.58 ± 2.68
17	3-Hydroxymandelate	5.55 ± 0.57	42.1 ± 9.71
18	3-Hydroxyphenylacetate	9.6 ± 6.2	50.05 ± 17.26
19	3-Indoxylsulfate	2.24 ± 2.24	13.06 ± 13.79
20	3-Methyladipate	46.48 ± 46.48	455.96 ± 315.28
21	3-Methylglutarate	7.13 ± 6.3	85.07 ± 166.22
22	3-Methylxanthine	1.05 ± 0.25	0.15 ± 0.02
23	3-Phenylpropionate	383.7 ± 104.3	214.4 ± 73.33
24	4-Hydroxy-3-methoxymandelate	2.08 ± 2.08	16.52 ± 17.96
25	4-Hydroxyphenylacetate	8.05 ± 1.56	33.9 ± 4.48
26	4-Pyridoxate	1.95 ± 0.34	0.36 ± 0.03
27	5-Aminolevulinate	4.08 ± 4.08	4.73 ± 5.38
28	5-Aminopentanoate	67.17 ± 0.25	203.97 ± 157.92
29	5-Hydroxyindole-3-acetate	6.2 ± 3.17	29.65 ± 4.32
30	5-Hydroxytryptophan	4.56 ± 4.56	30.07 ± 20.71
31	Acetaminophen	3.2 ± 2.56	8.8 ± 15.6
32	Acetate	27328.45 ± 3457.48	28365.9 ± 5109.27
33	Acetoacetate	11.3 ± 0.66	30.33 ± 22.56
34	Acetone	4.31 ± 4.66	0.95 ± 0.11
35	Acetylsalicylate	1.44 ± 1.44	24.35 ± 6.66
36	Alanine	27.25 ± 25.36	240.1 ± 17.34
37	Allantoin	4.29 ± 5.14	68.05 ± 99.93
38	Anserine	11.6 ± 7.07	73.95 ± 10.66
39	Arabinose	4.4 ± 4.4	56.85 ± 31.5

40	Betaine	1.96 ± 2.2	18.95 ± 3.57
41	Biotin	39.45 ± 32.35	128.1 ± 49.67
42	Butyrate	3319.4 ± 761.47	7613.7 ± 1319.69
43	Caffeine	3 ± 1.95	19.05 ± 6.36
44	Caprate	77.35 ± 17.97	235.53 ± 150.82
45	Carnitine	0.77 ± 0.61	11.91 ± 8.46
46	Carnosine	3.04 ± 3.04	28.3 ± 12.64
47	Cellobiose	11.39 ± 20.14	10.16 ± 14.37
48	Choline	1.96 ± 1.87	4.9 ± 1.35
49	Citrulline	26.96 ± 26.96	106.19 ± 107.83
50	Cytidine	2.08 ± 2.08	3.88 ± 5.68
51	Desaminotyrosine	5.06 ± 0.16	32.5 ± 5.15
52	Dimethyl sulfone	2.32 ± 2.71	6.45 ± 1.47
53	Dimethylamine	32.23 ± 27.07	17.79 ± 26.54
54	Erythritol	16.15 ± 5.12	73.08 ± 114.55
55	Ethanol	39.66 ± 23.61	402.29 ± 399.4
56	Ethylene glycol	4.23 ± 0.16	23.56 ± 14.36
57	Ferulate	2.01 ± 1.1	0.73 ± 0.5
58	Fructose	47.91 ± 41.99	136.61 ± 148.35
59	Fucose	4 ± 4	30.1 ± 34.79
60	Galactitol	11.02 ± 0.17	157.45 ± 69.27
61	Galactose	3.12 ± 3.12	154.4 ± 132.02
62	Gentisate	6.71 ± 5.74	1.88 ± 0.09
63	Gluconate	24.54 ± 23.04	6.15 ± 0.12
64	Glucose	126.75 ± 102.04	1608.5 ± 477.07
65	Glucose-6-phosphate	44.7 ± 25.48	275.71 ± 181.34
66	Glucuronate	11.04 ± 11.04	282.35 ± 90.25
67	Glutaric acid monomethyl ester	21.46 ± 0.67	67.2 ± 53.11
68	Glutathione	15.6 ± 15.6	31.75 ± 47.9
69	Glycylproline	41.9 ± 30.34	190.45 ± 81.67
70	Hippurate	2.96 ± 2.96	8.14 ± 7.93
71	Histamine	3.21 ± 1.9	27.11 ± 25.55
72	Histidine	2.4 ± 2.4	64.35 ± 23.61
73	Homovanillate	3.9 ± 2.17	35.75 ± 7.65
74	Hypoxanthine	2.18 ± 0.12	21.31 ± 17.25
75	Ibuprofen	5.12 ± 4.03	1.65 ± 0.13
76	Imidazole	19.45 ± 14.58	27.75 ± 8.78
77	Indole-3-acetate	4.65 ± 0.82	5.56 ± 5.64
78	Indole-3-lactate	1.96 ± 0.03	14.13 ± 10.16
79	Isobutyrate	95.78 ± 82.11	238.53 ± 255.8
80	Isocitrate	33.58 ± 20.24	247.33 ± 174.2
81	Isoleucine	9.05 ± 3.76	66.25 ± 36.03
82	Isopropanol	7.21 ± 5.66	43.55 ± 12.76

83	Isovalerate	60.76 ± 51.32	123.52 ± 137.59
84	Kynurenine	9.5 ± 2.96	36.3 ± 19.26
85	Lactose	46.39 ± 35.93	58.84 ± 54.34
86	Lactulose	25.1 ± 9.57	73.66 ± 63.58
87	Leucine	2.88 ± 2.88	48.4 ± 16.93
88	Malate	44.65 ± 12.8	81.81 ± 88.75
89	Maltose	47.25 ± 23.11	387.7 ± 221.96
90	Mandelate	1.52 ± 1.28	0.88 ± 0.88
91	Mannose	2.04 ± 0.04	9.17 ± 9.62
92	Melatonin	8.02 ± 4.88	11.85 ± 2.41
93	Methanol	20.75 ± 3.49	20.06 ± 19.18
94	Methionine	2.32 ± 2.71	15.23 ± 29.58
95	Methylamine	284.35 ± 50.79	126.1 ± 187.85
96	N,N-Dimethylformamide	1.12 ± 1.12	7.12 ± 13.12
97	N-Acetylcysteine	4.58 ± 4.33	56.55 ± 2.1
98	N-Acetylglucosamine	24.21 ± 17.11	189.16 ± 135.37
99	N-Acetylglutamate	7.63 ± 7.14	5.37 ± 6.82
100	N-Acetylglycine	19.9 ± 11.24	42.6 ± 49.87
101	N-Acetylornithine	11.43 ± 7.83	46.53 ± 46.76
102	N-Acetylserotonin	6.25 ± 2.18	4.39 ± 4.28
103	N-Acetyltyrosine	5.47 ± 6.22	13.19 ± 8.05
104	N-Carbamoylaspartate	33.2 ± 33.2	185.75 ± 32.65
105	N-Nitrosodimethylamine	8.9 ± 4.46	36.75 ± 6.29
106	N-Phenylacetylglycine	6.32 ± 5.33	31.21 ± 21.37
107	N-Phenylacetylphenylalanine	9.58 ± 7.42	16.01 ± 16.85
108	N6-Acetyllysine	21.52 ± 21.52	94.49 ± 55.99
109	NAD+	0.6 ± 0.03	3.9 ± 1.57
110	NADP+	0.74 ± 0.04	2.26 ± 1.78
111	NADPH	0.57 ± 0.07	3.63 ± 3.06
112	Niacinamide	2.17 ± 0.05	7.83 ± 6.9
113	Nicotinate	3.68 ± 0.22	25.25 ± 4.95
114	Nicotinurate	1.17 ± 0.05	10.8 ± 3.53
115	N-alpha-Acetyllysine	8.57 ± 7.36	101.75 ± 58.93
116	O-Acetylcarnitine	1.53 ± 1.94	20.15 ± 17.17
117	O-Acetylcholine	1.31 ± 2.07	6.13 ± 7.02
118	O-Phosphocholine	8.65 ± 5.95	40.85 ± 44.05
119	Oxypurinol	7.7 ± 4.26	0.89 ± 0.03
120	Pantothenate	6.16 ± 3.9	36.12 ± 55.18
121	Phenylacetate	16.45 ± 8.04	122.95 ± 81.51
122	Pimelate	53.32 ± 50.7	13.45 ± 0.09
123	Propionate	5919.45 ± 451.3	8813.9 ± 1173.33
124	Propylene glycol	4.4 ± 0.05	20.84 ± 11.9
125	Pyridoxine	0.99 ± 0.76	0.14 ± 0.01

126	Pyruvate	6.97 ± 5.85	2 ± 0.04
127	Riboflavin	1.85 ± 0.44	10.59 ± 11.9
128	Ribose	22.97 ± 17.96	597.1 ± 60.42
129	Salicylate	2.53 ± 0.04	14.45 ± 2.02
130	Salicylurate	5.26 ± 3.01	15.72 ± 17.94
131	Serotonin	6.25 ± 3.25	27.28 ± 18.12
132	Succinate	12.25 ± 4.24	1.52 ± 0.04
133	Succinylacetone	10.3 ± 4.48	1.4 ± 0.03
134	Sucrose	1.6 ± 1.6	65.8 ± 44.2
135	Syringate	2.25 ± 0.68	3.94 ± 5.05
136	Theophylline	0.85 ± 0.25	0.12 ± 0.01
137	Threonate	5.32 ± 4.29	12.37 ± 12.67
138	Thymol	5.7 ± 1.77	22.49 ± 22.43
139	Trehalose	2.96 ± 0.05	9.43 ± 7.52
140	Trimethylamine	2.43 ± 2.48	5.07 ± 9.02
141	Trimethylamine N-oxide	0.41 ± 0.05	1.5 ± 1.36
142	UDP-N-Acetylglucosamine	1.13 ± 0.89	0.4 ± 0.08
143	UDP-glucuronate	1.15 ± 0.11	3.41 ± 2.64
144	Uracil	6.86 ± 7.21	80.38 ± 53.47
145	Urea	25.68 ± 0.32	152.96 ± 103.7
146	Urocanate	2.4 ± 2.4	10.7 ± 8.28
147	Valerate	214.95 ± 125.09	2066.85 ± 300.82
148	Valine	13.87 ± 0.19	70.91 ± 40.87
149	Vanillate	1.13 ± 0.89	0.4 ± 0.03
150	Xanthine	4.72 ± 4.72	54.78 ± 60.7
151	Xanthosine	0.96 ± 0.96	1.26 ± 1.56
152	Xanthurenate	4.55 ± 4.16	10.25 ± 13.01
153	Xylitol	2.64 ± 2.64	190.9 ± 98.66
154	Xylose	4.32 ± 4.32	134.35 ± 96.75
155	cis-Aconitate	0.96 ± 0.96	7.25 ± 2.52
156	o-Cresol	7.44 ± 7.71	56.45 ± 25.22
157	p-Cresol	10.5 ± 2.08	19.51 ± 12.82
158	trans-Aconitate	4.53 ± 2.98	11.93 ± 9.01
159	1-Methylhistidine	4.75 ± 2.29	56.25 ± 42.62
160	3-Methylhistidine	2.38 ± 1.28	56.85 ± 7.84

[1)] NCD, normal concentrate diet (10 kg; Italian ryegrass 80 %: concentrate 20%)

[2)] HCD, high concentrate diet (14.2 kg; Italian ryegrass 20 %: concentrate 80%)
