# Supplementary material for: Comparison of metabolites in rumen fluid, urine, and feces of dairy cow from subacute ruminal acidosis model measured by proton nuclear magnetic resonance spectroscopy
Source: Anim Biosci. 2022 Aug 27;36(1):53–62. doi: 10.5713/ab.22.0124 (PMC9834661; doi:10.5713/ab.22.0124)
Supplement: Supplementary file 3 [file ab-22-0124-suppl3.pdf]

Supplementary table 3. Concentrations of the urine metabolite by ^{1}H-NMR analysis (μM, Median ± interquartile range, n = 4)

No.	Metabolite	NCD[1)]	HCD[2)]
1	1,3-Dihydroxyacetone	0.26 ± 0.38	0.58 ± 0.48
2	1,3-Dimethylurate	1.49 ± 1.52	2.69 ± 3.37
3	1,6-Anhydro-beta-D-glucose	4.02 ± 4.16	4.11 ± 4.14
4	1,7-Dimethylxanthine	5.44 ± 6.55	14.58 ± 7.33
5	2-Furoylglycine	4.71 ± 5.01	4.41 ± 4.41
6	2-Hydroxy-3-methylvalerate	12.09 ± 12.09	84.82 ± 56.65
7	2-Hydroxybutyrate	27.7 ± 0.06	132.49 ± 145.25
8	2-Hydroxyisobutyrate	1.21 ± 1.27	4.92 ± 3.44
9	2-Hydroxyisocaproate	17.34 ± 24.12	67.08 ± 34.4
10	2-Hydroxyphenylacetate	8.37 ± 5.61	17.91 ± 12.46
11	2-Hydroxyvalerate	67.28 ± 60.19	176.96 ± 156.79
12	2-Methylglutarate	5.32 ± 5.32	73.62 ± 47.35
13	3,4-Dihydroxybenzeneacetate	1.05 ± 1.05	15.15 ± 9.85
14	3,4-Dihydroxymandelate	4.82 ± 5.31	3.58 ± 6.01
15	3,5-Dibromotyrosine	60.49 ± 63.22	29.68 ± 44.55
16	3-Chlorotyrosine	6.7 ± 5.8	10.48 ± 17.17
17	3-Hydroxy-3-methylglutarate	9.23 ± 14.95	32.13 ± 20.99
18	3-Hydroxybutyrate	12.49 ± 0.01	262.86 ± 268.96
19	3-Hydroxyisovalerate	5.61 ± 5.95	22.74 ± 13.7
20	3-Hydroxymandelate	6.58 ± 4.8	15.36 ± 20.68
21	3-Hydroxyphenylacetate	20.1 ± 9.7	27.18 ± 2.74
22	3-Indoxylsulfate	39.39 ± 39.82	99.33 ± 76.95
23	3-Methyl-2-oxovalerate	7.1 ± 7.1	13.97 ± 20.84
24	3-Methylglutarate	110.58 ± 59.65	26.98 ± 21.83
25	3-Methylxanthine	1.23 ± 0.64	2.43 ± 1.51
26	3-Phenylpropionate	21.9 ± 23.85	65.49 ± 31.71
27	4-Aminohippurate	22.56 ± 37.59	7.53 ± 7.53
28	4-Guanidinobutanoate	3.54 ± 0.05	20.29 ± 13.28
29	4-Hydroxy-3-methoxymandelate	1.86 ± 1.11	11.94 ± 12.11
30	4-Hydroxyphenylacetate	66.81 ± 50.86	175.11 ± 142.25
31	4-Hydroxyphenyllactate	3.12 ± 0.02	19.63 ± 25.5
32	4-Pyridoxate	8.88 ± 10.44	8.97 ± 7.12
33	5-Aminolevulinate	10.47 ± 10.51	7.2 ± 3.58
34	5-Hydroxyindole-3-acetate	22.82 ± 23.85	44.94 ± 16.53
35	5-Hydroxytryptophan	3.63 ± 0.01	12.8 ± 11
36	5-Methoxysalicylate	1.57 ± 1.66	11.35 ± 19.28
37	Acetate	224.71 ± 202.85	93.56 ± 184.66
38	Acetoacetate	15.01 ± 16.99	125.1 ± 184.45
39	Acetoin	6.47 ± 5.28	6.03 ± 8.13

40	Acetone	2.68 ± 2.43	2.14 ± 1.8
41	Acetylsalicylate	21.87 ± 31.9	8.85 ± 4.96
42	Alanine	21.24 ± 14.5	162.99 ± 85.53
43	Allantoin	1267.8 ± 1189.1	2888.04 ± 1227.48
44	Anserine	5.67 ± 1.47	11.34 ± 7.52
45	Arabinose	9.3 ± 14.23	6.66 ± 7.66
46	Ascorbate	20.22 ± 16.97	10.44 ± 7.01
47	Betaine	43.35 ± 42.39	9.36 ± 6.43
48	Biotin	32.76 ± 24.68	4.8 ± 4.8
49	Butanone	14.79 ± 23.82	11.04 ± 10.2
50	Caffeine	2.67 ± 4.24	10.14 ± 3.71
51	Carnitine	0.24 ± 0.24	4.29 ± 1.27
52	Carnosine	15.26 ± 18.52	11.97 ± 9.96
53	Cellobiose	4.76 ± 4.76	83.49 ± 42.09
54	Cholate	3.1 ± 3.07	43.26 ± 35.11
55	Choline	1.08 ± 1.32	6.57 ± 4.05
56	Citraconate	1.09 ± 0.89	0.34 ± 0.01
57	Citrate	46.98 ± 42.89	25.98 ± 20.13
58	Creatine	6.84 ± 3.87	759.51 ± 898.69
59	Creatine phosphate	12.6 ± 6.09	52.8 ± 6.68
60	Creatinine	450.12 ± 484.76	940.08 ± 628.6
61	Desaminotyrosine	22.88 ± 17.9	29.08 ± 30.07
62	Dimethyl sulfone	10.87 ± 12.91	72.5 ± 61.41
63	Dimethylamine	103.75 ± 153.31	175.05 ± 216.28
64	Ethylene glycol	3.31 ± 3.31	39.27 ± 18.34
65	Ethylmalonate	8.64 ± 9.51	7.77 ± 7.77
66	Ferulate	3.31 ± 5.08	0.78 ± 0.47
67	Formate	77.19 ± 64.98	189.18 ± 68.05
68	Fructose	60.05 ± 80.41	72.21 ± 46.46
69	Fucose	26.4 ± 14.35	53.94 ± 20.55
70	Fumarate	0.42 ± 0.36	2.46 ± 0.84
71	Galactarate	41.4 ± 38.62	34.74 ± 14.21
72	Galactitol	26.24 ± 41.54	22.05 ± 21.24
73	Galactonate	6.94 ± 11.2	55.98 ± 48.18
74	Gallate	160.68 ± 131.13	29.1 ± 18.88
75	Gentisate	35.32 ± 66.31	10.87 ± 19.87
76	Glucarate	11.18 ± 11.18	39.34 ± 31.79
77	Gluconate	59.44 ± 89.17	79.79 ± 50.72
78	Glucose-6-phosphate	43.76 ± 74.07	63.23 ± 83.83
79	Glucuronate	36.43 ± 52.41	71.61 ± 59.77
80	Glutaric acid monomethyl ester	7.05 ± 13.62	11.94 ± 9.93
81	Glycine	2.92 ± 2.92	93.87 ± 78.89
82	Glycocholate	0.45 ± 0.02	14.26 ± 16

83	Glycolate	3331.92 ± 2471.57	1127.01 ± 1263.23
84	Glycylproline	134.79 ± 116.85	41.55 ± 35.22
85	Guanidoacetate	16.45 ± 9.41	82.98 ± 65.14
86	Hippurate	10061.22 ± 6287.04	3525.63 ± 1611.28
87	Histamine	22.23 ± 26.67	24.57 ± 21.51
88	Histidine	10.32 ± 10.92	26.34 ± 23.68
89	Homogentisate	4.59 ± 6.78	7.8 ± 10.25
90	Homovanillate	22.08 ± 30.71	5.52 ± 4.45
91	Hydroxyacetone	0.67 ± 0.67	3.36 ± 1.17
92	Ibuprofen	8.13 ± 8.73	1.2 ± 1.2
93	Imidazole	11.19 ± 7.71	14.67 ± 7.03
94	Indole-3-acetate	44.37 ± 29.58	51.69 ± 14.01
95	Indole-3-lactate	23.14 ± 24.55	35.43 ± 13.51
96	Isocitrate	107.16 ± 126.9	187.68 ± 140.75
97	Isoeugenol	3.16 ± 3.16	5.83 ± 8.5
98	Isoleucine	11.57 ± 12.08	6.37 ± 10.12
99	Kynurenate	6.1 ± 6.41	2.71 ± 2.55
100	Kynurenine	12 ± 0.19	47.58 ± 45.22
101	Lactose	515.04 ± 357.51	489.69 ± 411.71
102	Lactulose	14.64 ± 16.93	59.82 ± 26.72
103	Levulinate	0.9 ± 0.04	16.38 ± 11.08
104	Malate	26.68 ± 24.05	7.16 ± 0.07
105	Malonate	14.7 ± 12.28	24.09 ± 7.86
106	Maltose	18.67 ± 14.82	101.05 ± 113.6
107	Mandelate	22.59 ± 20.41	33.69 ± 15.32
108	Mannose	3.87 ± 4.77	7.67 ± 8.86
109	Melatonin	17.03 ± 32.72	13.27 ± 20.83
110	Methanol	26.46 ± 13.36	12.55 ± 11.69
111	Methylamine	7.44 ± 4.53	9.9 ± 6.1
112	Methylguanidine	0.47 ± 0.02	1.66 ± 1.48
113	Methylsuccinate	28.17 ± 17.36	11.73 ± 11.2
114	N,N-Dimethylformamide	2.75 ± 2.7	5.21 ± 5.14
115	N,N-Dimethylglycine	0.87 ± 0.43	1.47 ± 1.91
116	N-Acetylaspartate	0.69 ± 0.15	10.59 ± 17.87
117	N-Acetylglucosamine	6.89 ± 9.75	2.63 ± 2.22
118	N-Acetylglutamate	2.36 ± 2.66	8.91 ± 13.16
119	N-Acetylglutamine	2.84 ± 0.02	21.17 ± 16.12
120	N-Acetylserotonin	14.31 ± 12.94	14.7 ± 4.06
121	N-Acetyltyrosine	5.56 ± 6.56	1.31 ± 1.34
122	N-Carbamoyl-beta-alanine	10.34 ± 0.02	31.97 ± 24.98
123	N-Isovaleroylglycine	2.78 ± 0.02	9.1 ± 7.39
124	N-Methylhydantoin	7.62 ± 5.38	4.8 ± 3.71
125	N-Nitrosodimethylamine	32.71 ± 49.36	77.28 ± 72.38

126	N-Phenylacetylglycine	171.18 ± 101.04	1080.24 ± 503.79
127	N6-Acetyllysine	20.98 ± 19.51	26.16 ± 12.1
128	Niacinamide	3.92 ± 5.3	5.94 ± 6.42
129	Nicotinurate	1.26 ± 0.02	7.87 ± 5.47
130	O-Acetylcarnitine	1.72 ± 2.68	2.07 ± 1.33
131	O-Acetylcholine	1.61 ± 1.82	2.35 ± 2.45
132	O-Phosphocholine	1.82 ± 1.89	2.11 ± 2.78
133	Ornithine	14.62 ± 16.48	38.62 ± 48.95
134	Oxypurinol	1.9 ± 1.73	3.87 ± 0.57
135	Pantothenate	18.84 ± 12.62	19.71 ± 17.32
136	Phenylacetate	14.43 ± 13.17	22.02 ± 10.1
137	Propylene glycol	8.76 ± 0.04	46.95 ± 27.85
138	Pyridoxine	2.7 ± 3.15	10.93 ± 12.76
139	Pyruvate	0.15 ± 0.03	1.22 ± 1.69
140	Riboflavin	3.51 ± 1.81	4.11 ± 1.98
141	Ribose	19.83 ± 33.27	28.02 ± 31.41
142	Salicylate	13.11 ± 20.74	1.11 ± 0.11
143	Salicylurate	10.49 ± 8.19	7.26 ± 7.71
144	Sarcosine	2.07 ± 0.03	15.87 ± 9.14
145	Sebacate	93.2 ± 120.29	40.4 ± 57.86
146	Serotonin	11.93 ± 10.29	30.68 ± 27.72
147	Succinate	5.82 ± 3.63	10.8 ± 10.33
148	Succinylacetone	32.04 ± 29.31	17.1 ± 15.11
149	Sucrose	6.76 ± 6.76	25.44 ± 16.56
150	Syringate	165.3 ± 117.37	31.74 ± 59.11
151	Tartrate	0.58 ± 0.64	0.52 ± 0.52
152	Taurine	25.54 ± 0.03	163.98 ± 141.33
153	Theophylline	1.17 ± 1.47	2.4 ± 2.9
154	Thymidine	2.22 ± 1.82	0.64 ± 0.04
155	Thymol	19.83 ± 12.91	8.79 ± 4.03
156	Trehalose	0.62 ± 0.62	0.89 ± 1.16
157	Trimethylamine	0.6 ± 0.88	1.53 ± 1.13
158	Trimethylamine N-oxide	3663.6 ± 1769.9	924.57 ± 429.14
159	Tryptophan	63.15 ± 39.37	39.71 ± 50.72
160	Tyramine	3.26 ± 3.26	5.93 ± 8.6
161	Tyrosine	17.3 ± 17.2	87.82 ± 68.7
162	UDP-glucose	1.77 ± 1.77	2.88 ± 3.99
163	UMP	4.29 ± 6.18	2.4 ± 2.4
164	Urea	5165.97 ± 3153.81	23295.69 ± 12539.73
165	Valerate	46.2 ± 69.75	22.65 ± 22.65
166	Valine	4.12 ± 4.69	3.55 ± 3.55
167	Vanillate	118.23 ± 81.05	3.27 ± 2.29
168	Xanthine	170.64 ± 119.68	56.37 ± 19.72

169	Xanthurenate	47.34 ± 25.18	27.15 ± 13.3
170	Xylose	7.84 ± 9.01	30.86 ± 25.9
171	cis-Aconitate	28.29 ± 24.38	41.7 ± 33.41
172	o-Cresol	10.23 ± 10.72	23.46 ± 16.54
173	p-Cresol	7.77 ± 4.66	8.16 ± 6.66
174	sn-Glycero-3-phosphocholine	0.84 ± 0.87	2.41 ± 1.77
175	trans-Aconitate	6.99 ± 3.54	4.56 ± 3.06
176	1-Methylhistidine	64.11 ± 107.96	8.28 ± 2.27
177	3-Methylhistidine	43.47 ± 30.91	42.24 ± 18.49

[1)] NCD, normal concentrate diet (10 kg; Italian ryegrass 80 %: concentrate 20%)

[2)] HCD, high concentrate diet (14.2 kg; Italian ryegrass 20 %: concentrate 80%)
