# Supplementary material for: Comparison of metabolites in rumen fluid, urine, and feces of dairy cow from subacute ruminal acidosis model measured by proton nuclear magnetic resonance spectroscopy
Source: Anim Biosci. 2022 Aug 27;36(1):53–62. doi: 10.5713/ab.22.0124 (PMC9834661; doi:10.5713/ab.22.0124)
Supplement: Supplementary file 4 [file ab-22-0124-suppl4.pdf]

Supplementary table 4. Concentrations of the feces metabolite by ^{1}H-NMR analysis (μM, Median ± interquartile range, n = 4)

No.	Metabolite	NCD[1)]	HCD[2)]
1	1,3-Dihydroxyacetone	0.69 ± 0.85	2.47 ± 1.69
2	1,3-Dimethylurate	0.15 ± 0.01	1.23 ± 1.31
3	1,7-Dimethylxanthine	0.67 ± 0.7	2.32 ± 0.87
4	2'-Deoxyuridine	1.38 ± 0.07	4.68 ± 3.99
5	2-Aminobutyrate	4.86 ± 6.06	10.51 ± 12.95
6	2-Hydroxy-3-methylvalerate	8.74 ± 9.68	15.92 ± 17.98
7	2-Hydroxyisobutyrate	0.75 ± 0.38	0.55 ± 0.67
8	2-Hydroxyisocaproate	6.48 ± 12.07	15.86 ± 27.94
9	2-Hydroxyisovalerate	0.6 ± 0.6	3.45 ± 4.07
10	2-Hydroxyphenylacetate	4.57 ± 0.71	7.31 ± 2.82
11	2-Hydroxyvalerate	22.86 ± 31.15	42.84 ± 34.42
12	2-Oxocaproate	1.28 ± 0.11	8.97 ± 6.25
13	2-Oxoisocaproate	0.61 ± 0.51	0.99 ± 0.96
14	3,4-Dihydroxybenzeneacetate	1.34 ± 1.22	5.13 ± 6.29
15	3,5-Dibromotyrosine	0.51 ± 0.66	0.36 ± 0.36
16	3-Hydroxy-3-methylglutarate	2.88 ± 4.41	12.45 ± 8.25
17	3-Hydroxybutyrate	1.16 ± 0.09	7.17 ± 6.08
18	3-Hydroxyisovalerate	1.05 ± 0.7	5.32 ± 4.36
19	3-Hydroxymandelate	6.88 ± 4.2	8.77 ± 3.77
20	3-Hydroxyphenylacetate	6.78 ± 0.94	8.25 ± 2.76
21	3-Indoxylsulfate	1.14 ± 1.14	4.11 ± 4.38
22	3-Methyl-2-oxovalerate	0.28 ± 0.05	0.96 ± 0.83
23	3-Methylglutarate	9.75 ± 9.18	32.63 ± 53.01
24	3-Methylxanthine	0.47 ± 0.28	0.78 ± 0.14
25	3-Phenylpropionate	13.85 ± 8.84	38.55 ± 24.11
26	4-Aminobutyrate	2.46 ± 2.46	6.16 ± 6.84
27	4-Hydroxy-3-methoxymandelate	1.08 ± 1.09	4.5 ± 3.46
28	4-Hydroxyphenylacetate	7.76 ± 6.46	12.71 ± 7.41
29	4-Hydroxyphenyllactate	11.57 ± 10.84	2.97 ± 0.09
30	4-Pyridoxate	1.53 ± 1.55	2.58 ± 2.1
31	5-Aminolevulinate	7.05 ± 4.11	24.3 ± 20.53
32	5-Hydroxyindole-3-acetate	2.13 ± 1.74	3.37 ± 1.35
33	5-Hydroxylysine	3.91 ± 0.11	16.61 ± 16.72
34	5-Hydroxytryptophan	4.92 ± 4.8	4.67 ± 2.56
35	5-Methoxysalicylate	2.73 ± 1.9	0.76 ± 0.76
36	Acetamide	0.55 ± 0.08	28.52 ± 31.33
37	Acetaminophen	2.32 ± 1.19	2.53 ± 3.58
38	Acetate	3983.73 ± 2861.36	6399.41 ± 2774.5
39	Acetoacetate	1.62 ± 1.27	1.44 ± 1.15

40	Acetone	2.25 ± 1.7	0.71 ± 0.82
41	Acetylsalicylate	0.96 ± 1.75	3.15 ± 3.68
42	Allantoin	1.81 ± 1.89	1.74 ± 1.74
43	Alloisoleucine	1.34 ± 2.2	3.27 ± 5.43
44	Anserine	3.3 ± 1.41	5.62 ± 4
45	Arabinose	5.88 ± 5.06	39.63 ± 23.32
46	Azelate	22.26 ± 21.45	5.53 ± 0.06
47	Benzoate	2.64 ± 2.64	6.13 ± 6.12
48	Betaine	2.22 ± 3.63	2.67 ± 3.45
49	Biotin	6.5 ± 7.06	22.49 ± 17.14
50	Butanone	2.91 ± 1.68	17.7 ± 7.23
51	Butyrate	283.91 ± 285.51	497.81 ± 334.56
52	Caffeine	1.23 ± 1.09	2.4 ± 1.57
53	Caprate	8.34 ± 8.34	40.34 ± 24.75
54	Carnitine	0.42 ± 0.42	0.45 ± 0.49
55	Carnosine	2.73 ± 2.52	3.41 ± 2.24
56	Cellobiose	10.98 ± 12.5	1.62 ± 1.62
57	Choline	4.42 ± 3.03	2.24 ± 2.25
58	Citrate	1.89 ± 1.56	0.57 ± 0.02
59	Creatine	5.01 ± 9.36	9.37 ± 12.77
60	Creatine phosphate	0.93 ± 0.92	1.91 ± 1.29
61	Creatinine	4.95 ± 3.61	5.36 ± 1.64
62	Cytidine	1.86 ± 1.86	5.74 ± 3.55
63	Desaminotyrosine	2.31 ± 2.35	4.2 ± 3.76
64	Dimethyl sulfone	1.38 ± 1.4	2.7 ± 1.47
65	Dimethylamine	0.31 ± 0.22	1.62 ± 1.52
66	Erythritol	12.51 ± 13.35	22.27 ± 8.22
67	Ethanol	29.92 ± 12.45	15.98 ± 10.58
68	Ethylene glycol	1.01 ± 0.65	1.16 ± 0.76
69	Ferulate	1.91 ± 0.6	1.4 ± 0.8
70	Formate	24.15 ± 5.15	24.07 ± 7.8
71	Fructose	19.45 ± 16.59	20.39 ± 12.68
72	Fucose	13.28 ± 15.11	21.03 ± 8.11
73	Galactarate	6.18 ± 2.63	3.87 ± 3.87
74	Galactitol	8.52 ± 5.25	6.98 ± 3.76
75	Galactonate	14.63 ± 9.15	26.81 ± 20.38
76	Galactose	33.77 ± 25.15	44.88 ± 33.44
77	Gentisate	1.45 ± 1.53	2.1 ± 1.63
78	Glucarate	0.74 ± 0.05	4.3 ± 2.74
79	Glucitol	19.11 ± 18.05	37.7 ± 23.91
80	Gluconate	17.26 ± 15.78	12.91 ± 10.26
81	Glucose	71.47 ± 30.36	117.52 ± 39.54
82	Glucose-6-phosphate	20.22 ± 20.34	27.96 ± 19.17

83	Glucuronate	14.33 ± 15.94	29.83 ± 21.7
84	Glutamate	18.19 ± 15.26	62.96 ± 20.75
85	Glutarate	11.56 ± 15.39	32.39 ± 24.99
86	Glutaric acid monomethyl ester	3.81 ± 3.5	2.73 ± 2.3
87	Glutathione	6.09 ± 4.75	6.43 ± 8.91
88	Glycine	12.48 ± 12.48	20.25 ± 21.42
89	Glycolate	5.92 ± 7.48	2.25 ± 2.21
90	Glycylproline	7.23 ± 9.4	42 ± 38.45
91	Guanidoacetate	4.12 ± 2.56	5.51 ± 3.48
92	Hippurate	0.9 ± 0.02	3.67 ± 3.57
93	Histamine	2.13 ± 1.11	3.67 ± 1.63
94	Histidine	0.6 ± 0.05	4.5 ± 1.34
95	Homocystine	3.93 ± 4.08	9.49 ± 10.77
96	Homogentisate	3.46 ± 2.82	0.87 ± 1.02
97	Homovanillate	3.07 ± 0.58	6.71 ± 4.2
98	Hydroxyacetone	1.59 ± 1.59	5.81 ± 3.07
99	Ibuprofen	1.8 ± 1.15	3.23 ± 3.9
100	Imidazole	4.57 ± 1.08	11.1 ± 5.02
101	Indole-3-acetate	1.38 ± 1.38	2.85 ± 2.76
102	Indole-3-lactate	1.26 ± 1.26	2.04 ± 2.83
103	Isobutyrate	95.28 ± 41.45	130.57 ± 76.32
104	Isocitrate	31.2 ± 7.79	49.38 ± 32.54
105	Isoeugenol	2.34 ± 1.31	2.94 ± 2.63
106	Isoleucine	5.7 ± 3.1	10.53 ± 4.99
107	Isopropanol	1.9 ± 2.23	5.02 ± 1.98
108	Isovalerate	54.6 ± 21.13	86.32 ± 40.74
109	Kynurenate	0.51 ± 0.04	1.77 ± 1.44
110	Kynurenine	3.09 ± 4.74	6.86 ± 2.18
111	Lactose	19.1 ± 11.81	54.54 ± 42.89
112	Lactulose	10.74 ± 11.77	24.33 ± 25.22
113	Leucine	9.15 ± 5.32	13.31 ± 6.68
114	Levulinate	3.33 ± 3.14	2.19 ± 3.84
115	Malonate	2.42 ± 1.63	3.9 ± 0.61
116	Mandelate	1.17 ± 1.08	0.75 ± 0.6
117	Mannose	9.87 ± 7.04	12.68 ± 12.15
118	Melatonin	3.46 ± 2.89	4.31 ± 2.71
119	Methanol	22.12 ± 19.39	37.35 ± 21.73
120	Methionine	2.83 ± 1.72	9.9 ± 5.61
121	Methylamine	1.16 ± 1.2	0.6 ± 0.57
122	Methylguanidine	5.1 ± 2.59	2.31 ± 2.08
123	Methylsuccinate	3.59 ± 3.33	1.4 ± 2.26
124	N,N-Dimethylformamide	0.8 ± 0.83	2.07 ± 2.48
125	N,N-Dimethylglycine	1.41 ± 1.56	2.45 ± 2.66

126	N-Acetylaspartate	0.96 ± 0.76	2.73 ± 3.37
127	N-Acetylcysteine	3.07 ± 3.11	7.87 ± 7.88
128	N-Acetylglucosamine	15.86 ± 8.05	13.38 ± 8.22
129	N-Acetylglutamate	3.03 ± 3.22	6.69 ± 4.52
130	N-Acetylglutamine	1.44 ± 1.44	2.52 ± 3.61
131	N-Acetylglycine	8.02 ± 7.36	26.17 ± 27.15
132	N-Acetylornithine	2.8 ± 3.93	7.78 ± 6.23
133	N-Acetylserotonin	2.3 ± 1.58	2.26 ± 1.47
134	N-Acetyltyrosine	3.24 ± 3.24	7.34 ± 3.98
135	N-Isovaleroylglycine	0.24 ± 0.24	1.56 ± 1.73
136	N-Methylhydantoin	0.95 ± 1.06	2.72 ± 3.57
137	N-Nitrosodimethylamine	3.93 ± 2.12	8.1 ± 3.18
138	N-Phenylacetylglycine	3.45 ± 2.99	6.07 ± 5.78
139	N-Phenylacetylphenylalanine	3.36 ± 3.36	6.99 ± 6.64
140	N6-Acetyllysine	3.74 ± 5.13	12.9 ± 15.21
141	Nicotinate	1.52 ± 0.77	3.27 ± 2.82
142	N-alpha-Acetyllysine	0.9 ± 0.12	7.76 ± 6.67
143	O-Acetylcarnitine	2.81 ± 4.02	0.96 ± 0.81
144	O-Acetylcholine	2.51 ± 1.38	2.04 ± 1.61
145	O-Phosphocholine	2.68 ± 1.54	2.57 ± 1.51
146	Pantothenate	3.11 ± 1.71	6.26 ± 5.44
147	Phenylacetate	10.61 ± 4.2	9.85 ± 8.57
148	Propionate	736.42 ± 617.73	1193.1 ± 610.27
149	Propylene glycol	1.62 ± 0.08	5.64 ± 4.67
150	Pyridoxine	0.6 ± 0.57	0.93 ± 0.68
151	Pyruvate	1.08 ± 1.21	8.85 ± 9.36
152	Riboflavin	0.54 ± 0.41	1.13 ± 0.63
153	Ribose	35.31 ± 41.38	54.22 ± 35.04
154	Salicylurate	1.05 ± 0.04	6.9 ± 4.81
155	Sarcosine	2.25 ± 2.49	3.82 ± 4.74
156	Serotonin	3.6 ± 3.6	7.05 ± 6.12
157	Succinate	6.37 ± 1.34	8.47 ± 1.15
158	Succinylacetone	1.23 ± 0.87	3.6 ± 2.68
159	Sucrose	0.72 ± 0.72	1.28 ± 1.84
160	Syringate	0.42 ± 0.45	0.34 ± 0.31
161	Tartrate	0.72 ± 0.7	0.76 ± 0.71
162	Theophylline	0.3 ± 0.25	0.56 ± 0.14
163	Threonate	4.32 ± 6.72	1.92 ± 1.92
164	Threonine	5.01 ± 4.35	14.09 ± 18.29
165	Thymol	3.15 ± 1.56	5.21 ± 2.24
166	Trehalose	9.18 ± 4.36	8.73 ± 10.17
167	Trimethylamine N-oxide	0.86 ± 1.23	0.82 ± 0.77
168	Tryptophan	1.26 ± 1.26	3.09 ± 4.93

169	UDP-N-Acetylglucosamine	0.3 ± 0.3	1.05 ± 0.82
170	Urea	20.1 ± 21.79	33.55 ± 29.72
171	Uridine	1.38 ± 1.08	4.16 ± 2.88
172	Urocanate	1.2 ± 1.2	4.12 ± 2.48
173	Valerate	75.36 ± 88	217.61 ± 98.31
174	Valine	8.93 ± 6.13	13.27 ± 12.17
175	Xanthine	3.4 ± 2.65	5.41 ± 3.1
176	Xanthurenate	6.22 ± 3.61	8.36 ± 1.53
177	Xylitol	9.08 ± 10	15.88 ± 11.26
178	Xylose	25.68 ± 17.06	22.27 ± 14.19
179	cis-Aconitate	1.86 ± 1.86	5.3 ± 5.07
180	dTTP	0.72 ± 0.08	2.58 ± 2.19
181	o-Cresol	2.81 ± 2.66	10.35 ± 9.54
182	p-Cresol	1.65 ± 1.58	4.68 ± 2.35
183	sn-Glycero-3-phosphocholine	3.75 ± 2.02	1.1 ± 1.66
184	trans-Aconitate	1.68 ± 2.24	3.41 ± 1.71
185	beta-Alanine	0.76 ± 0.12	10.77 ± 17.97
186	gamma-Glutamylphenylalanine	6.25 ± 6.63	5.88 ± 5.88
187	1-Methylhistidine	2.77 ± 0.75	5.02 ± 2.97
188	3-Methylhistidine	1.38 ± 1.2	2 ± 1.28

[1)] NCD, normal concentrate diet (10 kg; Italian ryegrass 80 %: concentrate 20%)

[2)] HCD, high concentrate diet (14.2 kg; Italian ryegrass 20 %: concentrate 80%)
